# Bone Microarchitecture and Strength Changes During Teriparatide and Zoledronic Acid Treatment in a Patient with Pregnancy and Lactation-Associated Osteoporosis with Multiple Vertebral Fractures

**DOI:** 10.1007/s00223-023-01066-3

**Published:** 2023-02-10

**Authors:** Sanne Treurniet, Melissa S. A. M. Bevers, Caroline E. Wyers, Dimitra Micha, Bernd P. Teunissen, Mariet W. Elting, Joop P. van den Bergh, Elisabeth M. W. Eekhoff

**Affiliations:** 1 Department of Internal Medicine Section Endocrinology, Rare Bone Disease Center, Amsterdam, Movement Sciences, Amsterdam UMC, location Vrije Universiteit Amsterdam, Amsterdam, The Netherlands; 2 Department of Internal Medicine, VieCuri Medical Center, Venlo, The Netherlands; 3 NUTRIM School for Nutrition and Translational Research in Metabolism, Maastricht University Medical Center, Maastricht, The Netherlands; 4 Department of Biomedical Engineering, Eindhoven University of Technology, Eindhoven, The Netherlands; 5 Department of Internal Medicine, Subdivision of Rheumatology, Maastricht University Medical Center, Maastricht, The Netherlands; 6 Department of Human Genetics, Amsterdam Movement Sciences, Amsterdam Rare Bone Disease/Amsterdam Bone Center, Amsterdam University Medical Center, location Vrije Universiteit Amsterdam, De Boelelaan 1117, Amsterdam, The Netherlands; 7 Department of Radiology and Nuclear Medicine, Amsterdam UMC, location Vrije Universiteit Amsterdam, Amsterdam, The Netherlands

**Keywords:** Pregnancy- and lactation-associated osteoporosis, Teriparatide, Zoledronic acid, Bone microarchitecture, Bone strength, High-resolution peripheral quantitative computed tomography

## Abstract

Pregnancy- and lactation-associated osteoporosis (PLO) is a rare form of osteoporosis, of which the pathogenesis and best treatment options are unclear. In this report, we describe the case of a 34-year old woman diagnosed with severe osteoporosis and multiple vertebral fractures after her first pregnancy, who was subsequently treated with teriparatide (TPTD) and zoledronic acid (ZA). We describe the clinical features, imaging examination, and genetic analysis. Substantial improvements were observed in areal and volumetric bone mineral density (BMD), microarchitecture, and strength between 7 and 40 months postpartum as assessed by dual-energy X-ray absorptiometry at the total hip and spine and by high-resolution peripheral quantitative CT at the distal radius and tibiae. At the hip, spine, and distal radius, these improvements were mainly enabled by treatment with TPTD and ZA, while at the distal tibiae, physiological recovery and postpartum physiotherapy due to leg pain after stumbling may have played a major role. Additionally, the findings show that, despite the improvements, BMD, microarchitecture, and strength remained severely impaired in comparison with healthy age- and gender-matched controls at 40 months postpartum. Genetic analysis showed no monogenic cause for osteoporosis, and it is suggested that PLO in this woman could have a polygenic origin with possible susceptibility based on familiar occurrence of osteoporosis.

**Supplementary Information:**

The online version contains supplementary material available at 10.1007/s00223-023-01066-3.

## Introduction

Pregnancy and lactation-associated osteoporosis (PLO) is a rare form of osteoporosis, characterized by back pain and vertebral compression fractures, that affects women during pregnancy and early postpartum [1]. Hormonal-mediated adaptations to fulfill an increased calcium demand can cause physiological bone loss during pregnancy and lactation, while additional factors (e.g. genetics) may contribute to the pathological bone loss in PLO [2]. Although bone mineral density (BMD) restores spontaneously postweaning in most women, some present postpartum with persistent severe back pain and progressive height loss due to severe osteoporosis and vertebral fractures. In severe cases, treatment with anti-osteoporosis drugs can be considered although optimal treatment and pharmacological need are incompletely known [1]. In this case report, we describe a 34-year old woman diagnosed with multiple vertebral fractures after pregnancy and the effects of treatment with teriparatide (TPTD) and zoledronic acid (ZA) on bone microarchitecture and strength assessed with high-resolution peripheral quantitative CT (HR-pQCT).

## Case Presentation

A 34-year old woman presented in a local hospital with severe back pain. Seven months earlier, she gave birth to her first child after an uncomplicated pregnancy and delivery. During the second trimester of pregnancy, she had a period of heavy pain in her right leg after stumbling. Heavy back pain started during the third trimester. At the moment of presentation, she was breastfeeding her child. Her medical history revealed a herniated nucleus pulposus 6 years earlier, no previous fractures, and a regular menstrual cycle before pregnancy. Her father was diagnosed with osteoporosis at the age of 48 and her mother and sister with osteopenia. No other osteoporosis-related risk factors were present. X-ray examination, magnetic resonance imaging (MRI), and dual-energy X-ray absorptiometry (DXA) revealed multiple vertebral compression fractures (Th8 and Th12), height loss of all mid and low thoracic vertebra (quantification not available), lumbar spine end plate indentation, and osteoporosis (*T*-score lumbar spine: − 4.2; total hip: − 2.7). She had lost 4 cm of her best recalled height; her body mass index was normal (25 kg/m^2^). Laboratory tests showed normal biochemistries, hematology, and thyroid level, and a 25-hydroxy vitamin D level of 54 nmol/L (ref. > 50 nmol/L). Based on these findings, she was advised cessation of breastfeeding and supplementation of calcium and vitamin D.

**Fig. 1:**
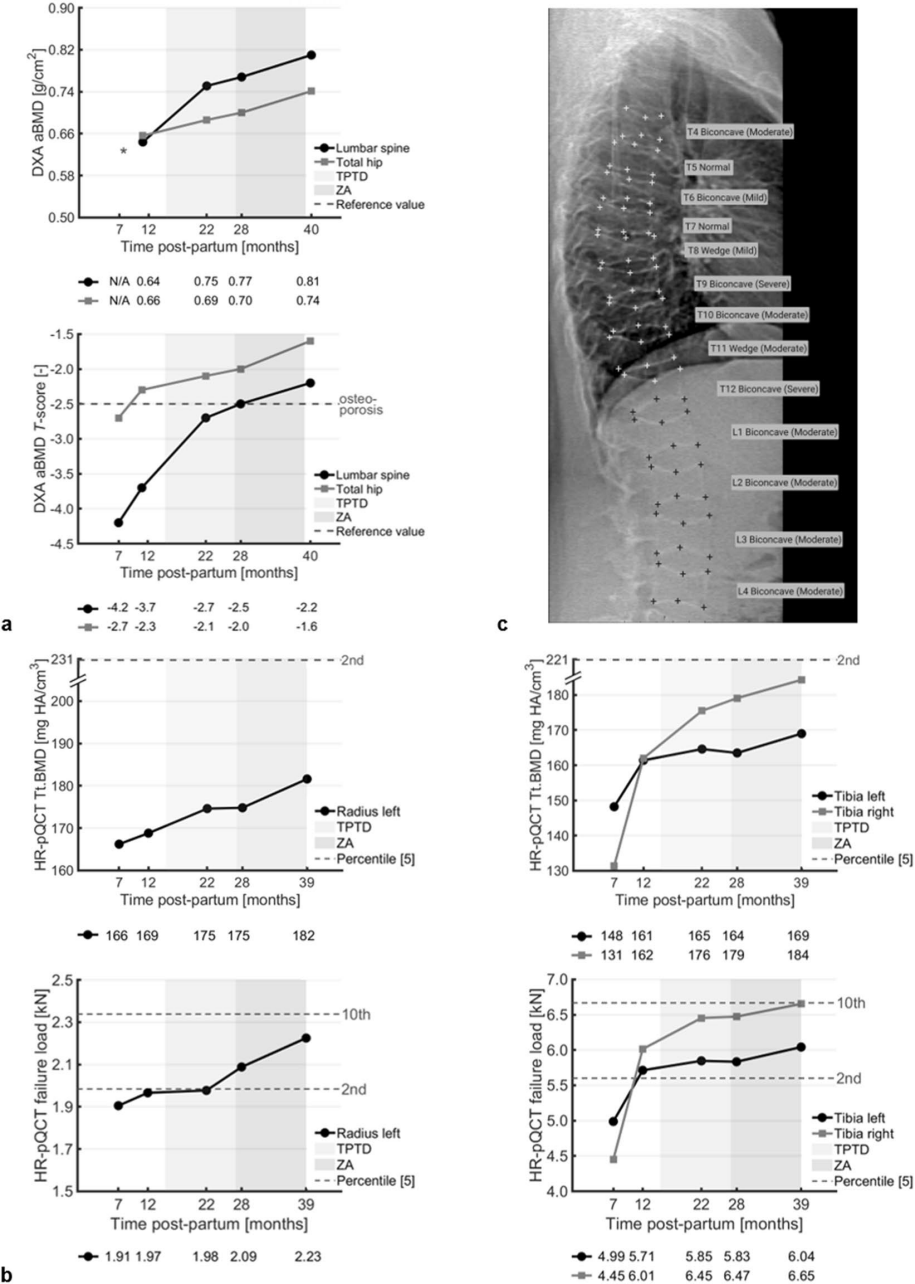
**a** Changes in areal BMD values (top) and *T*-scores (bottom) of the woman’s lumbar spine (black) and total hip (grey) from DXA examinations between 7 and 40 months postpartum. **b** Changes in total BMD (top) and failure load (bottom) at the woman’s left distal radius (left) and both distal tibiae (right) from HR-pQCT examinations between 7 and 39 months postpartum. **c** Instant vertebral assessment (IVA) of the woman’s spine from the DXA examination at 40 months postpartum. TPTD is teriparatide, ZA is zoledronic acid, * indicates not available, and the dotted grey lines indicate the *T*-score that defines osteoporosis (**a**) and the percentile scores from age- and gender-matched normative data (**b**) [5]

**Fig. 2:**
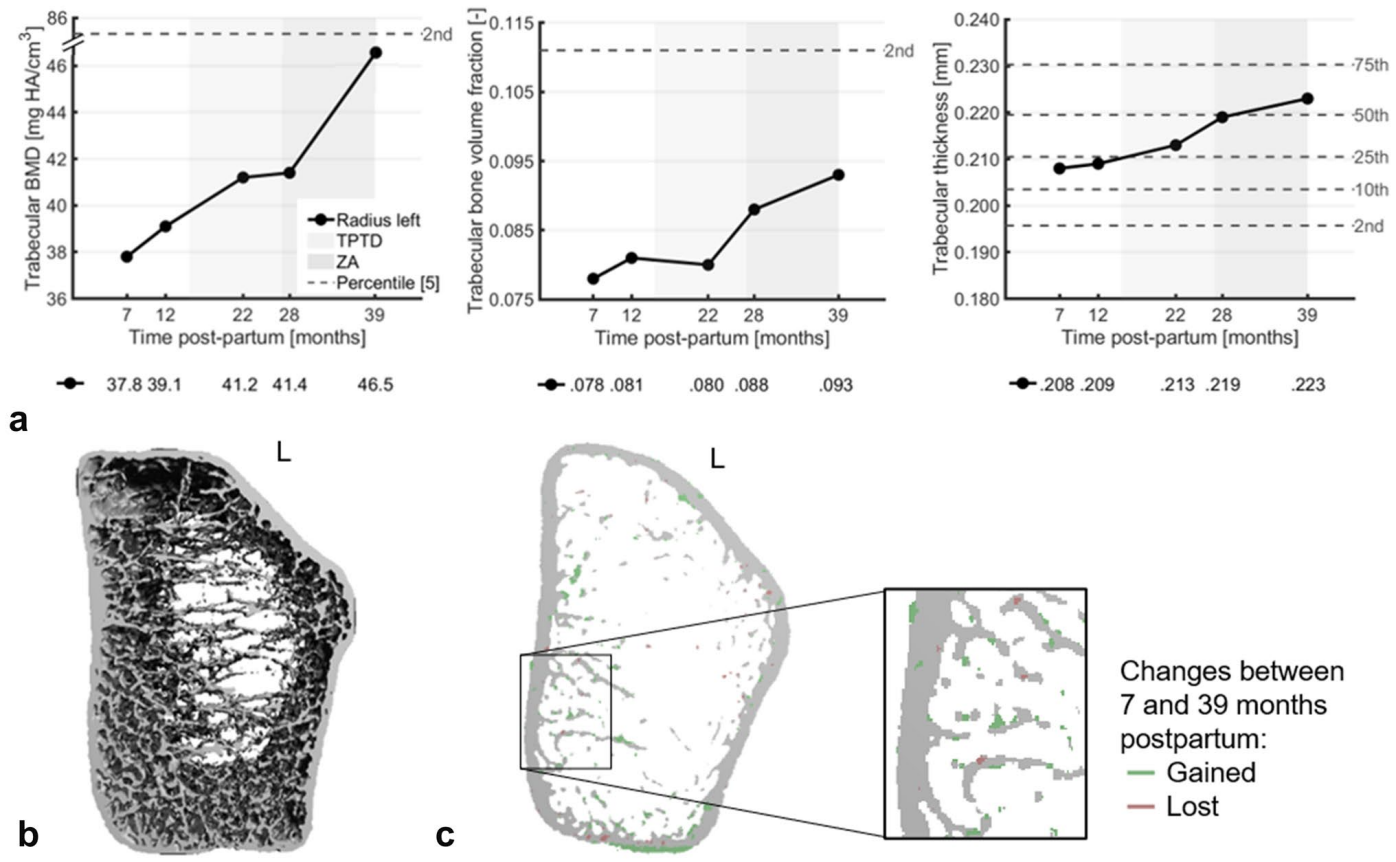
**a** Changes in parameters at the woman’s left distal radius from HR-pQCT examinations between 7 and 39 months postpartum that greatly exceeded least-significant changes from literature at 39 months. TPTD is teriparatide, ZA is zoledronic acid, and the dotted grey lines indicate percentile scores from age- and gender-matched normative data [5]. **b** Three-dimensional visualization of the HR-pQCT scan of the left distal radius at 7 months postpartum. **c** Axial mid-slice of the left distal radius showing bone gain (green) and loss (pink) between 7 and 39 months postpartum from HR-pQCT examinations

**Fig. 3:**
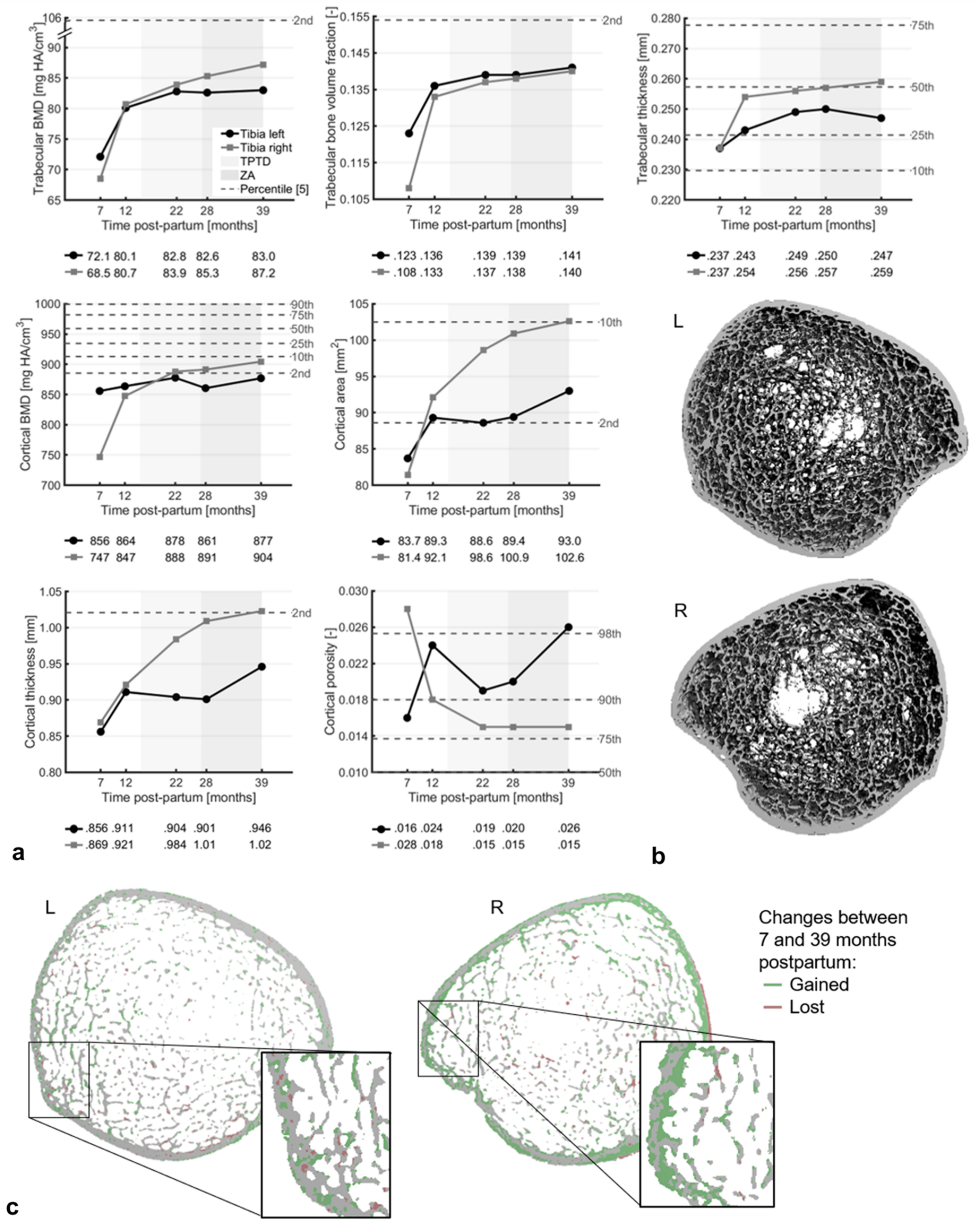
**a** Changes in trabecular parameters (top) and cortical parameters (middle, bottom) at the woman’s left (black) and right (grey) distal tibia from HR-pQCT examinations between 7 and 39 months postpartum that greatly exceeded least-significant changes from literature at 39 months. TPTD is teriparatide, ZA is zoledronic acid, and the dotted grey lines indicate percentile scores from age- and gender-matched normative data [5]. **b** Three-dimensional visualization of the HR-pQCT scan of the distal tibiae at 7 months postpartum. **c** Axial mid-slice of the distal tibiae showing bone gain (green) and loss (pink) between 7 and 39 months postpartum from HR-pQCT examinations

One month later, 8 months postpartum, she was referred to Amsterdam UMC outpatient clinic for further analysis. Next generation sequencing was performed with a panel including 19 osteoporosis and osteogenesis imperfecta related genes (Online Resource 1) [3], followed by whole exome sequencing (trio-analysis with DNA of her parents). Genetic and biochemical analyses revealed no monogenic mutation nor secondary causes of osteoporosis, so she was diagnosed with PLO. We advised her to continue calcium and vitamin D supplementation. Eleven months postpartum, DXA examination showed improvements in lumbar spine and total hip areal BMD (aBMD) (Fig. 1a) and HR-pQCT examination in volumetric BMD, bone microarchitecture, and strength (in terms of failure load) at the distal radius and distal tibiae (Figs. 1b, 2, 3, Online Resource 2, 3). Nevertheless, the woman experienced increased back pain, indicating progressive height loss of the vertebrae, and aBMD remained low (*T*-score lumbar spine: − 3.7; total hip: − 2.3). This worried her as she desired to have a second child. Therefore, 15 months postpartum, after shared decision-making, TPTD 20 µg/day was prescribed for 1 year followed by one dose of zoledronic acid (ZA). We advised her to postpone a second pregnancy until 1 year after the ZA-dose.

Three consecutive DXA and HR-pQCT examinations showed further improvements. Lumbar spine and total hip aBMD increased by 25.8% and 13.0%, respectively, between 11 and 40 months postpartum, resulting in *T*-scores in the osteopenic range (− 2.2 and − 1.6, respectively) (Fig. 1a). DXA vertebral fracture assessment showed multiple compression fractures, which were not further increased since the start of TPTD treatment (Fig. 1c). The distal radius showed increases between 7 and 39 months postpartum greatly exceeding least-significant changes (LSCs) from literature in total and trabecular BMD (+ 9.3% and + 23.2%, respectively), trabecular bone volume fraction and thickness (+ 19.8% and + 7.2%, respectively), and failure load (+ 16.8%) (Figs. 1b, 2a) [4]. Increases in all other parameters except cortical porosity were within several percentage points above or below LSCs (Online Resource 2). The distal tibiae also showed increases largely exceeding LSCs in total BMD (left tibia: + 14.0%; right tibia: + 40.2%), trabecular BMD (left: + 15.1%; right: + 27.3%), trabecular bone volume fraction (left: + 14.7%; right: + 29.2%), trabecular thickness (left: + 4.4%; right: + 9.2%), and failure load (left: + 21.1%; right: + 49.5%) and additionally in cortical BMD (left: + 2.5%; right: + 21.1%), area (left: + 11.2%; right: + 26.1%), thickness (left: + 10.6%; right: + 17.7%), and porosity (left: + 62.4%; right: − 45.0%) (Figs. 1b, 3a). Other changes were within percentage points around LSCs (Online Resource 3). Most parameters at 39 months postpartum remained nevertheless at the lowest 2–10 percentile of an age- and gender-matched normative dataset [5, 6].

## Discussion

Most women experience no or modest decreases in aBMD during pregnancy and larger decreases during lactation with spontaneous recovery postweaning [7]. Additionally, they may experience a decrease in volumetric BMD and changes in bone microarchitecture at the distal tibia and proximal radius [8, 9]. In PLO, bone loss is aggravated, and impaired volumetric BMD and bone microarchitecture have been reported compared to healthy controls [10] and healthy lactating women [11]. The woman in this report also had a severely impaired aBMD, volumetric BMD, and bone microarchitecture compared to healthy controls, which even after treatment persisted until 40 months postpartum.

In line with previous studies [7, 8, 10, 11], the trabecular bone was more affected than the cortical bone but recovered earlier. At 7 months postpartum, aBMD *T*-score was worse at the lumbar spine (predominantly trabecular) than at the total hip (cortical and trabecular), and trabecular BMD was relatively lower than cortical BMD at the distal radius and tibia. Until 40 months postpartum, lumbar spine aBMD showed larger increases than total hip aBMD, and trabecular BMD increased more at the radius and tibia than cortical BMD. The increase in trabecular BMD seemed the result of thickening of existing trabeculae and possibly the fusion of neighboring trabeculae. Nevertheless, a large increase was found in cortical BMD at the right tibia, possibly due to the filling of cortical pores and an increase in tissue mineralization. Despite the improvements, BMD and microarchitecture remained severely impaired at 40 months postpartum compared to healthy controls. This could have contributed to compensatory periosteal bone apposition in order to improve bone strength, as reflected by the changes in cortical and trabecular area and previously also suggested by Kovacs [7].

A possible factor in the pathological bone loss in PLO is heredity. Recent studies showed that multiple genetic mutations, in *LRP5* and *WNT1*, can aggravate bone loss in PLO [2, 10, 12, 13]. Although no monogenic cause was found in this woman, osteoporosis and osteopenia occur in her immediate family. Additionally, HR-pQCT scans of her mother’s distal radius and tibia showed similar cortical discrepancies (Online Resource 4). It suggests a polygenic origin in this patient with unknown contribution of both her parents’ side.

Currently, there is no clinical guideline for the management of patients with PLO, and treatment strategy remains challenging and controversial due to the lack of intervention-controlled research. Termination of breastfeeding is important but may not be sufficient, especially in case of multiple vertebral fractures [14]. Prolonged bisphosphonate treatment is suggested to be successful [15], but bisphosphonates accumulate in the skeleton and can be released in a subsequent pregnancy, possibly harming the fetus. TPTD does not accumulate in the body and has been found to increase aBMD, relieve pain, and prevent new vertebral fractures in PLO [16–18]. It also results in greater increases in aBMD compared to no TPTD treatment in PLO [19]. Interestingly, Lee et al. showed no significant difference in aBMD 3 years after discontinuation of TPTD treatment between PLO-patients with and without successive antiresorptive therapy [20]. It suggests that it may be advantageous not to start antiresorptive treatment after TPTD discontinuation when a PLO-patient desires to have children in the future. As this finding was not available when the woman presented at our hospital, we treated her with one dose of ZA after TPTD.

The largest improvements were generally seen during TPTD treatment and until 1 year after the ZA-dose, albeit site-dependent. At the distal radius, lumbar spine, and hip, the largest changes occurred during treatment. At the distal tibiae in contrast, they occurred before treatment. The latter may be due to spontaneous recovery and possibly also due to the physiotherapy that the woman followed after pregnancy because of right leg pain after stumbling during pregnancy. The stumbling and consequent disuse of the right leg for 5 months may also explain the worse HR-pQCT parameters at 7 months at the right tibia compared to the left tibia.

## Conclusion

This case report showed the follow-up of a woman with multiple vertebral fractures due to PLO without an identified monogenic cause. Treatment with teriparatide and zoledronic acid resulted in substantial improvements in bone mineral density, microarchitecture, and strength although larger improvements were observed before treatment at the tibiae. Nevertheless, bone quality remained considerably impaired compared to healthy controls at 40 months postpartum.

## Supplementary Information

Below is the link to the electronic supplementary material.

Supplementary file1 (PDF 123 KB)

Supplementary file2 (PDF 191 KB)

Supplementary file3 (PDF 200 KB)

Supplementary file4 (PDF 143 KB)
