# Supplementary material for: Bone Microarchitecture and Strength Changes During Teriparatide and Zoledronic Acid Treatment in a Patient with Pregnancy and Lactation-Associated Osteoporosis with Multiple Vertebral Fractures
Source: Calcif Tissue Int. 2023 Feb 10;112(5):621–7. doi: 10.1007/s00223-023-01066-3 (PMC10106348; doi:10.1007/s00223-023-01066-3)
Supplement: Supplementary file 1 — Supplementary file1 (PDF 123 KB) [file 223_2023_1066_MOESM1_ESM.pdf]

Online Resource 1:

Panel with osteoporosis and osteogenesis imperfecta related genes used for the next generation sequencing (NGS).

Online Resource Table 1.1: NGS panel with osteoporosis and osteogenesis imperfecta related genes
ALPL
BMP1
COL1A1
COL1A2
CREB3L1
CRTAP
FKBP10
IFITM5
LEPRE1
LRP5
PLOD2
PLS3
PPIB
SERPINF1
SERPINH1
SP7
TAPT1
TMEM38B
WNT1
