# Supplementary material for: Bone Microarchitecture and Strength Changes During Teriparatide and Zoledronic Acid Treatment in a Patient with Pregnancy and Lactation-Associated Osteoporosis with Multiple Vertebral Fractures
Source: Calcif Tissue Int. 2023 Feb 10;112(5):621–7. doi: 10.1007/s00223-023-01066-3 (PMC10106348; doi:10.1007/s00223-023-01066-3)
Supplement: Supplementary file 2 — Supplementary file2 (PDF 191 KB) [file 223_2023_1066_MOESM2_ESM.pdf]

Online Resource 2: Bone geometry, bone mineral density, bone microarchitecture, and strength at the distal radius of the women with PLO assessed with high-resolution peripheral quantitative CT (HR-pQCT).

Online Resource Table 2.1: Geometry, BMD, and microarchitecture at the left distal radius of the women with PLO assessed with HR-pQCT.

	**Visit 1 (7.3 mo PP)**	**Visit 2 (12.1 mo PP)**		**Visit 3 (22.0 mo PP)**		**Visit 4 (28.2 mo PP)**		**Visit 5 (39.9 mo PP)**		
	Value	Value	Change w.r.t visit 1 (%)	Value	Change w.r.t visit 1 (%)	Value	Change w.r.t visit 1 (%)	Value	Change w.r.t visit 1 (%)	Percentile score
Geometry										
Tb.Ar (mm^2)	244.3	244.1	-0.1	242.7	-0.6	242.9	-0.6	242.2	-0.8	75-90
Ct.Ar (mm^2)	44.1	44.1	+0.2	45.6	+3.4	45.3	+2.9	46.1	+4.6	2-10
Volumetric density										
Tt.BMD (mg HA/cm^3)	166.2	168.8	+1.6	174.6	+5.1	174.8	+5.2	181.6	+9.3	<2
Tb.BMD (mg HA/cm^3)	37.8	39.1	+3.5	41.2	+9.1	41.4	+9.6	46.5	+23.2	<2
Ct.BMD (mg HA/cm^3)	913.6	922.5	+1.0	917.8	+0.5	923.5	+1.1	923.9	+1.1	25-75
Microarchitecture										
Tb.BV/TV (-)	0.078	0.081	+3.9	0.080	+2.7	0.088	+13.4	0.093	+19.8	<2
Tb.N (mm^{-1})	0.498	0.504	+1.2	0.468	-5.9	0.476	-4.4	0.479	-3.7	<2
Tb.Th (mm)	0.208	0.209	+0.6	0.213	+2.8	0.219	+5.5	0.223	+7.2	25-75
Tb.Sp (mm)	2.030	2.001	-1.5	2.185	+7.6	2.126	+4.7	2.122	+4.5	<2*
Tb.1/N.SD (mm)	1.727	1.682	-2.6	1.767	+2.3	1.793	+3.8	1.813	+4.9	<2*
Ct.Th (mm)	0.735	0.727	-1.0	0.751	+2.2	0.746	+1.6	0.761	+3.6	2-10
Ct.Po (-)	0.002	0.002	+15.7	0.001	-29.0	0.002	+16.3	0.002	-1.9	25-75*

Tt: total, Tb: trabecular, Ct: cortical, Ar: area, BMD: bone mineral density, BV/TV: bone volume fraction, N: number, Th: thickness, Sp: separation, 1/N.SD: heterogeneity, Po: porosity.

Parameter values were obtained from the overlapping portions of the radius on the HR-pQCT scans between the visits after slice-matching (92% of 168 slices). The time between brackets indicates months postpartum at each visit. * indicates parameters for which the percentile scores are reversed (*e.g.* a score of <2 represents a value larger, and thus worse, than the 98th percentile). Percentile scores are according to the normative dataset of Whittier DE, et al. 2020 J Bone Miner Res 35:2151-2158.

Online Resource Table 2.2: Strength at the left distal radius of the women with PLO estimated with micro-finite element analysis from HR-pQCT.

	**Visit 1 (7.3 mo PP)**	**Visit 2 (12.1 mo PP)**		**Visit 3 (22.0 mo PP)**		**Visit 4 (28.2 mo PP)**		**Visit 5 (39.9 mo PP)**		
	Value	Value	Change w.r.t visit 1 (%)	Value	Change w.r.t visit 1 (%)	Value	Change w.r.t visit 1 (%)	Value	Change w.r.t visit 1 (%)	Percentile score
Strength										
Failure load (kN)	1.905	1.966	+3.2	1.977	+3.8	2.088	+9.6	2.225	+16.8	2-10

Parameter values were obtained from the entire HR-pQCT scans without registration of the HR-pQCT scans between the visits. The time between brackets indicates months postpartum at each visit. Percentile scores are according to the normative dataset of Whittier DE, et al. 2020 J Bone Miner Res 35:2151-2158.
