# Supplementary material for: Bone Microarchitecture and Strength Changes During Teriparatide and Zoledronic Acid Treatment in a Patient with Pregnancy and Lactation-Associated Osteoporosis with Multiple Vertebral Fractures
Source: Calcif Tissue Int. 2023 Feb 10;112(5):621–7. doi: 10.1007/s00223-023-01066-3 (PMC10106348; doi:10.1007/s00223-023-01066-3)
Supplement: Supplementary file 3 — Supplementary file3 (PDF 200 KB) [file 223_2023_1066_MOESM3_ESM.pdf]

Online Resource 3:

Bone geometry, bone mineral density, bone microarchitecture, and strength at the distal tibiae of the women with PLO assessed with high-resolution peripheral quantitative CT (HR-pQCT).

Online Resource Table 3.1: Geometry, BMD, and microarchitecture at the left distal tibia of the women with PLO of the assessed with HR-pQCT.

	**Visit 1 (7.3 mo PP)**	**Visit 2 (12.1 mo PP)**		**Visit 3 (22.0 mo PP)**		**Visit 4 (28.2 mo PP)**		**Visit 5 (39.9 mo PP)**		
	Value	Value	Change w.r.t visit 1 (%)	Value	Change w.r.t visit 1 (%)	Value	Change w.r.t visit 1 (%)	Value	Change w.r.t visit 1 (%)	Percentile score
Geometry										
Tb.Ar (mm^2)	747.9	742.2	-0.8	743.0	-0.6	742.2	-0.8	738.5	-1.2	90-98
Ct.Ar (mm^2)	83.7	89.3	+6.7	88.6	+5.9	89.4	+6.8	93.0	+11.2	2-10
Volumetric density										
Tt.BMD (mg HA/cm^3)	148.2	161.4	+8.9	164.6	+11.1	163.5	+10.3	169.0	+14.0	<2
Tb.BMD (mg HA/cm^3)	72.1	80.1	+11.1	82.8	+14.8	82.6	+14.6	83.0	+15.1	<2
Ct.BMD (mg HA/cm^3)	855.8	863.6	+0.9	877.7	+2.6	860.5	+0.6	876.9	+2.5	<2
Microarchitecture										
Tb.BV/TV (-)	0.123	0.136	+10.5	0.139	+13.6	0.139	+13.6	0.141	+14.7	<2
Tb.N (mm^{-1})	0.882	0.887	+0.6	0.879	-0.4	0.882	±0.0	0.882	±0.0	<2
Tb.Th (mm)	0.237	0.243	+2.5	0.249	+5.0	0.250	+5.6	0.247	+4.4	25-75
Tb.Sp (mm)	1.102	1.079	-2.1	1.095	-0.6	1.096	-0.6	1.084	-1.6	<2*
Tb.1/N.SD (mm)	0.494	0.482	-2.5	0.530	+7.3	0.525	+6.4	0.510	+3.3	<2*
Ct.Th (mm)	0.856	0.911	+6.4	0.904	+5.6	0.901	+5.3	0.946	+10.6	<2
Ct.Po (-)	0.016	0.024	+53.7	0.019	+17.9	0.020	+26.4	0.026	+62.4	<2*

Tt: total, Tb: trabecular, Ct: cortical, Ar: area, BMD: bone mineral density, BV/TV: bone volume fraction, N: number, Th: thickness, Sp: separation, 1/N.SD: heterogeneity, Po: porosity.

Parameter values were obtained from the overlapping portions of the tibiae on the HR-pQCT scans between the visits after slice-matching (95% of 168 slices). The time between brackets indicates months postpartum at each visit. * indicates parameters for which the percentile scores are reversed (*e.g.* a score of <2 represents a value larger, and thus worse, than the 98th percentile). Percentile scores are according to the normative dataset of Whittier DE, et al. 2020 J Bone Miner Res 35:2151-2158.

Online Resource Table 3.2: Geometry, BMD, and microarchitecture at the right distal tibia of the women with PLO assessed with HR-pQCT.

	**Visit 1 (7.3 mo PP)**	**Visit 2 (12.1 mo PP)**		**Visit 3 (22.0 mo PP)**		**Visit 4 (28.2 mo PP)**		**Visit 5 (39.9 mo PP)**		
	Value	Value	Change w.r.t visit 1 (%)	Value	Change w.r.t visit 1 (%)	Value	Change w.r.t visit 1 (%)	Value	Change w.r.t visit 1 (%)	Percentile score
Geometry										
Tb.Ar (mm^2)	758.1	747.7	-1.4	741.3	-2.2	739.3	-2.5	737.4	-2.7	90-98
Ct.Ar (mm^2)	81.4	92.1	+13.2	98.6	+21.2	100.9	+23.9	102.6	+26.1	10-25
Volumetric density										
Tt.BMD (mg HA/cm^3)	131.4	162.0	+23.2	175.5	+33.5	179.1	+36.3	184.3	+40.2	<2
Tb.BMD (mg HA/cm^3)	68.5	80.7	+17.8	83.9	+22.6	85.3	+24.5	87.2	+27.3	<2
Ct.BMD (mg HA/cm^3)	746.6	847.4	+13.5	888.0	+18.9	891.2	+19.4	904.4	+21.1	2-10
Microarchitecture										
Tb.BV/TV (-)	0.108	0.133	+22.5	0.137	+26.1	0.138	+26.9	0.140	+29.2	<2
Tb.N (mm^{-1})	0.816	0.798	-2.1	0.792	-2.9	0.787	-3.5	0.783	-4.0	<2
Tb.Th (mm)	0.237	0.254	+7.4	0.256	+7.9	0.257	+8.4	0.259	+9.2	25-75
Tb.Sp (mm)	1.204	1.221	+1.4	1.228	+2.0	1.244	+3.3	1.244	+3.4	<2*
Tb.1/N.SD (mm)	0.560	0.689	+23.2	0.752	+34.3	0.768	+37.1	0.813	+45.2	<2*
Ct.Th (mm)	0.869	0.921	+6.0	0.984	+13.2	1.009	+16.0	1.023	+17.7	2-10
Ct.Po (-)	0.028	0.018	-34.0	0.015	-48.0	0.015	-48.0	0.015	-45.0	10-25*

Tt: total, Tb: trabecular, Ct: cortical, Ar: area, BMD: bone mineral density, BV/TV: bone volume fraction, N: number, Th: thickness, Sp: separation, 1/N.SD: heterogeneity, Po: porosity.

Parameter values were obtained from the overlapping portions of the tibiae on the HR-pQCT scans between the visits after slice-matching (98% of 168 slices). The time between brackets indicates months postpartum at each visit. * indicates parameters for which the percentile scores are reversed (*e.g.* a score of <2 represents a value larger, and thus worse, than the 98th percentile). Percentile scores are according to the normative dataset of Whittier DE, et al. 2020 J Bone Miner Res 35:2151-2158.

Online Resource Table 3.3: Strength at the left and right distal tibiae of the women with PLO estimated with micro-finite element analysis from HR-pQCT.

	**Visit 1 (7.3 mo PP)**	**Visit 2 (12.1 mo PP)**		**Visit 3 (22.0 mo PP)**		**Visit 4 (28.2 mo PP)**		**Visit 5 (39.9 mo PP)**		
	Value	Value	Change w.r.t visit 1 (%)	Value	Change w.r.t visit 1 (%)	Value	Change w.r.t visit 1 (%)	Value	Change w.r.t visit 1 (%)	Percentile score
Failure load (kN)										
Distal tibia left	4.988	5.713	+14.5	5.847	+17.2	5.834	+17.0	6.042	+21.1	2-10
Distal tibia right	4.451	6.014	+35.1	6.453	+45.0	6.473	+45.4	6.653	+49.5	2-10

Parameter values were obtained from the entire HR-pQCT scans without registration of the HR-pQCT scans between the visits. The time between brackets indicates months postpartum at each visit. Percentile scores are according to the normative dataset of Whittier DE, et al. 2020 J Bone Miner Res 35:2151-2158.
