# Supplementary material for: Bone Microarchitecture and Strength Changes During Teriparatide and Zoledronic Acid Treatment in a Patient with Pregnancy and Lactation-Associated Osteoporosis with Multiple Vertebral Fractures
Source: Calcif Tissue Int. 2023 Feb 10;112(5):621–7. doi: 10.1007/s00223-023-01066-3 (PMC10106348; doi:10.1007/s00223-023-01066-3)
Supplement: Supplementary file 4 — Supplementary file4 (PDF 143 KB) [file 223_2023_1066_MOESM4_ESM.pdf]

Online Resource 4:

Bone geometry, BMD, and microarchitecture at the left distal radius and tibia of the mother of the women with PLO assessed with high-resolution peripheral quantitative CT (HR-pQCT).

Online Resource Table 4.1: Geometry, BMD, and microarchitecture at the left distal radius and tibia of the mother of the women with PLO assessed with HR-pQCT.

	**Distal radius**		**Distal tibia**	
	Value	Percentile score	Value	Percentile score
Geometry				
Tb.Ar (mm^2)	308.7	≥98	899.4	≥98
Ct.Ar (mm^2)	42.3	2-10	76.0	<2
Volumetric density				
Tt.BMD (mg HA/cm^3)	191.7	<2	152.1	<2
Tb.BMD (mg HA/cm^3)	116.0	25-75	106.6	2-10
Ct.BMD (mg HA/cm^3)	782.7	<2	719.3	<2
Microarchitecture				
Tb.BV/TV (-)	0.159	25-75	0.164	10-25
Tb.N (mm^{-1})	1.217	25-75	1.139	25-75
Tb.Th (mm)	0.223	25-75	0.238	10-25
Tb.Sp (mm)	0.805	25-75*	0.855	25-75*
Tb.1/N.SD (mm)	0.317	25-75*	0.327	25-75*
Ct.Th (mm)	0.614	<2	0.750	<2
Ct.Po (-)	0.004	75-90*	0.022	25-75*

Tt: total, Tb: trabecular, Ct: cortical, Ar: area, BMD: bone mineral density, BV/TV: bone volume fraction, N: number, Th: thickness, Sp: separation, 1/N.SD: heterogeneity, Po: porosity.

* indicates parameters for which the percentile scores are reversed (*e.g.* a score of <2 represents a value larger, and thus worse, than the 98th percentile). Percentile scores are according to the normative dataset of Whittier DE, et al. 2020 J Bone Miner Res 35:2151-2158.
